# Unraveling Molecular Signatures of Immunostimulatory Adjuvants in the Female Genital Tract through Systems Biology

**DOI:** 10.1371/journal.pone.0020448

**Published:** 2011-06-07

**Authors:** Madelene Lindqvist, Intawat Nookaew, Ingrid Brinkenberg, Emma Samuelson, Karolina Thörn, Jens Nielsen, Ali M. Harandi

**Affiliations:** 1 Department of Microbiology and Immunology, Institute of Biomedicine, Sahlgrenska Academy, University of Gothenburg, Gothenburg, Sweden; 2 Department of Chemical and Biological Engineering, Chalmers University of Technology, Gothenburg, Sweden; Tulane University, United States of America

## Abstract

Sexually transmitted infections (STIs) unequivocally represent a major public health concern in both industrialized and developing countries. Previous efforts to develop vaccines for systemic immunization against a large number of STIs in humans have been unsuccessful. There is currently a drive to develop mucosal vaccines and adjuvants for delivery through the genital tract to confer protective immunity against STIs. Identification of molecular signatures that can be used as biomarkers for adjuvant potency can inform rational development of potent mucosal adjuvants. Here, we used systems biology to study global gene expression and signature molecules and pathways in the mouse vagina after treatment with two classes of experimental adjuvants. The Toll-like receptor 9 agonist CpG ODN and the invariant natural killer T cell agonist alpha-galactosylceramide, which we previously identified as equally potent vaginal adjuvants, were selected for this study. Our integrated analysis of genome-wide transcriptome data determined which signature pathways, processes and networks are shared by or otherwise exclusive to these 2 classes of experimental vaginal adjuvants in the mouse vagina. To our knowledge, this is the first integrated genome-wide transcriptome analysis of the effects of immunomodulatory adjuvants on the female genital tract of a mammal. These results could inform rational development of effective mucosal adjuvants for vaccination against STIs.

## Introduction

Although the vast majority of human pathogens invade the body and/or establish infections in the mucosal tissues, only a handful of mucosal vaccines are currently licensed for human use. Sexually transmitted infections (STIs) are a major public health concern in both industrialized and developing countries. Despite numerous efforts, the human papillomavirus vaccines Gardasil® and Cervarix®, given by intramuscular injection, represent the only human vaccines licensed to prevent an STI. Previous attempts to develop vaccines for systemic immunization against other sexually transmitted pathogens in humans have failed [1], [2]. This failure has prompted a great interest in developing vaccines and immunomodulators for delivery via mucosal routes, including the female genital tract, to confer protective mucosal immunity to sexually transmitted pathogens. While sexually transmitted pathogens generate pathogen-specific immune responses, local administration of non-replicating antigens into the vagina engenders little to no protective mucosal immune response [3], [4]. To overcome this hurdle, immunologic adjuvants with the ability to mount mucosal immune responses with co-administered vaccine antigens to confer immunity in the female genital tract are desirable. However, no mucosal adjuvants are currently available for human use [5], [6]. We have previously shown that the Toll-like receptor 9 (TLR9) agonist CpG ODN and the invariant natural killer T (NKT) cell agonist α-galactosylceramide (α-GalCer) can function as potent vaginal adjuvants when administered together with herpes simplex virus type 2 (HSV-2) glycoprotein D, giving rise to comparable protective immunity against genital HSV-2 infection and disease in mice [7], [8]. Rational design of effective and safe mucosal adjuvants for human use requires a thorough understanding of the mode of action of promising candidate adjuvants in addition to identification of biomarkers that predict their potency.

Previous efforts to comprehend the effect of immunologic adjuvants on genome-wide gene expression were performed in isolated immune cells stimulated with CpG ODN *in vitro* [9], [10] or muscles and spleens isolated from mice after intramuscular or intra-peritoneal injection with CpG ODN, alum or MF59 [11], [12]. Using real time RT-PCR, we have recently shown that CpG ODN and α-GalCer induce the gene expression of several cytokines and chemokines in the murine female genital tract [13]. However, there is a dearth of information on the impact of mucosal administration of promising experimental adjuvants on global gene expression in the female genital tract.

To address this issue, we employed a genome-wide transcript microarray analysis combined with a systems biology approach. This approach involved an integrated analysis of transcriptome data to comprehend the molecular correlates of adjuvant efficacy in the murine female genital tract after treatment with two classes of experimental mucosal adjuvants, CpG ODN and α-GalCer. This approach offers the unique advantage of monitoring the structure and dynamics of multifaceted cell-to-cell interactions *in vivo*.

We were able to show that vaginal administration of CpG ODN generated a rapid induction in the expression of a large number of genes in the murine vagina between 4 h and 48 h after adjuvant delivery. α-GalCer, however, created a delayed and transient gene expression induction. The expression of 343 genes was commonly up-regulated in the vagina after vaginal delivery of CpG ODN or α-GalCer. Integrated bio-functionality analyses identified different pathways involved in innate immune responses by the two adjuvants. However, “inflammatory response” was found as the main bio-function induced by both CpG ODN and α-GalCer, and IFN-γ was identified as the main interactor of the bio-function “inflammatory response” shared by both adjuvants. In addition, analyses of the kinetics of the gene expression revealed highly interconnected genes that may play important roles in adjuvant efficacy.

This study showed that 2 classes of candidate vaginal adjuvants, CpG ODN and α-GalCer, share molecular signatures that may contribute to their adjuvant efficacy in the female genital tract. To our knowledge, this is the first report that identifies genome-wide molecular correlates for adjuvant efficacy in the mammalian female genital tract.

## Materials and Methods

### Animal experiments

The study was approved by the Ethical Committee for Animal Experimentation in Gothenburg, Swedish Animal Welfare Authority (djurskyddsmyndigheten) with permit number 267-09. Eight-week-old female C57Bl/6 mice (Charles River, Germany) were used for this study. The mice were kept under specific pathogen-free conditions in IVC cages with unlimited access to food and water and under constant humidity, temperature and a 12-h dark/light cycle at the Experimental Biomedicine Animal Facility, Sahlgrenska Academy, at the University of Gothenburg. Isoflourane (Baxter Medical AB) was used to anesthetize the mice. To synchronize estrus cycles, all mice were injected subcutaneously with 3 mg of Depo-Provera (DP, Pfizer), a long-lasting progestin, in 150 µl of sterile phosphate-buffered saline (PBS). CpG ODN 1826 (TCC ATG ACG TTC CTG ACG TT, hereafter CpG ODN), a 20-mer containing two copies of optimal mouse CpG ODN motifs with complete phosphorothioate backbones, was purchased from Operon Biotechnologies GMBH, Germany and reconstituted in PBS. α-GalCer (Alexis Biochemicals) was stored in chloroform methanol 2∶1 (vol/vol) and after evaporation, reconstituted in PBS/Tween 0.5% prior to use. Six days after DP treatment, mice were intravaginally (i.vag.) administered with a single dose of either CpG ODN (30 µg) or α-GalCer (5 µg) in 19 µl. These reagent doses were previously shown to function as potent vaginal adjuvants. Together with HSV-2 gD antigen, they induced comparable levels of protective immunity in the mouse vagina against genital herpes infection [7], [8]. Control groups were given the same volume of the PBS or PBS/Tween 0.5% reagent diluents i.vag. Whole mouse vaginas (cut right below the cervix) were aseptically removed at different time points after vaginal administration of the adjuvants and subjected to microarray analysis, cytokine assessment of the tissue extract or flow cytometric analysis, as explained below.

### Genome wide expression microarray

Whole mouse vaginas were collected in RNAlater™ (QIAGEN GmbH, Hilden, Germany) at 4 h, 24 h and 48 h after vaginal administration. Total RNA was extracted using the RNeasy mini kit (QIAGEN) following the manufacturer's protocol with an extra DNase elimination step during the last wash using the RNase-Free DNase kit (Qiagen). RNA quality was assessed with an Agilent 2100 BioAnalyzer (Agilent Technologies, Paolo Alto, CA) and visualized on a 2% agarose gel. Only good quality RNA samples were used for further processing. Two hundred nanograms of each sample were prepared and hybridized to Affymetrix Mouse Gene 1.0 ST arrays according to Affymetrix's recommended protocol (GeneChip® Whole Transcript (WT) Sense Target Labeling Assay, 701880 Rev. 5). Hybridization and analysis were performed according to the manufacturer's instructions at the SCIBLU Genomics core facility (Swegene Centre for Integrative Biology at Lund University, Sweden).

### Data acquisition and analysis of transcriptome data

We first evaluated the intrinsic variability in the transcriptome data. Briefly, raw intensity files (CEL) were normalized and processed together, and both Probe Logarithmic Intensity Error (PLIER) with iterative algorithm [14] and quantile normalization [15] were chosen to identify the expression signal. The intrinsic variability of the data was evaluated by singular value decomposition (SVD), and first three left Eigen arrays were illustrated in a pseudo-three dimensional plot (the third dimension is represented by the dot size). Significant differences in gene expression between CpG ODN and PBS and between α-GalCer and PBS/Tween 0.5% at each given time point after treatments, i.e., 4 h, 24 h and 48 h, were evaluated using Student's *t*-test. The calculated p-values were transformed into Q-values by correcting for multiple testing using methods described by Benjamini and Hochberg [16]. To identify cellular processes and adaptations in response to the treatments, statistical Q-values were mapped in the context of Gene Ontology (GO) biological networks and pathways [17]–[18] using a reporter algorithm [19], [20]. Q-values, log 2 fold changes, the pathway and GO networks including their associated gene members used for the integration analysis is provided in Table S1. P-values≤0.001 from the reporter algorithm were used in hierarchical clustering to determine the pattern of responses, and the results are illustrated as a heat map of significance values. Gene co-expression analyses for each treatment were performed on groups of genes with Q-values of≤0.01 for at least one time point. Co-expression modules were identified by weighted gene co-expression network analysis (WGCNA) [21]. The results are shown as a heat map of topological overlap matrices that indicate co-expression modules. Functional enrichment of each identified module was further evaluated by modular enrichment analysis [22]. All analyses were performed using R suite (http://www.R-project.org) and Cytoscape software [23].

### Accession codes

All data are MIAME compliant and were deposited in the MIAME compliant database National Center for Biotechnology Information Gene Expression Omnibus (GEO; http://www.ncbi.nlm.nih.gov/geo/) and are available through the GEO with accession number GSE27149.

### Gene network analysis

The immune gene network was analyzed with Ingenuity Pathway Analysis (IPA; Ingenuity® Systems Inc., Redwood City, CA, USA, www.ingenuity.com). IPA maps each gene within a global molecular network developed from information contained in the Ingenuity Pathways Knowledge Base. Gene networks are generated algorithmically based on their connectivity in terms of expression, activation, transcription, and/or inhibition. An IPA “network” is defined as a graphical representation of the molecular relationships between genes, represented as nodes, and biological relationships, represented as connecting lines between nodes. All connections are supported by published data stored in the Ingenuity Pathways Knowledge Base and/or PubMed for mice. IPA ranks all genes based on their connectivity, using a generalization of the node degree concept, which measures the number of other genes to which a gene is connected. IPA-based analysis has been successfully employed to elucidate relationships and connections between differentially expressed genes. For example, IPA elucidated the mechanisms of action of CpG ODN in mouse spleens in addition to the mechanism of action of the human yellow fever vaccine [11], [24].

### Assessment of cytokine proteins in the mouse vagina

Vaginas were collected at 4 h and 8 h after vaginal administration of adjuvants. Tissue samples were placed immediately in PBS containing 1.5 mM Pefabloc SC (Charles River Laboratories Endosafe), 0.1 mg/ml soybean trypsin inhibitor (Sigma), 0.05 M EDTA and 1% BSA and frozen at −70°C after weighing. Tissue samples were thawed and permeabilized overnight at 4°C in PBS with 2% (w/v) saponin (Sigma Aldrich). Samples were centrifuged for 10 min at 12,000 rpm, and supernatants were collected. Vaginal extracts were analyzed for IL-1β using a cytokine ELISA kit (Duoset, R&D kit) according to the manufacturer's recommendations. TNF-α, IFN-γ, IL-10 and IL-6 protein levels were analyzed using a mouse inflammation CBA kit (BD Biosciences) on a BD FACSCalibur, according to the manufacturer's instructions for BD CBA software. During data acquisition, a gate was set around polystyrene beads. Data were analyzed using BD CBA software, and standard curves were made using 4-parameter logistic curve fitting, from which cytokine concentrations were calculated. GraphPad Prism 4 software (GraphPad Software, USA) was used for statistical analysis of protein expression. Results are shown as the mean+standard error of mean (SEM). Statistical significance of the variance between multiple groups was calculated with one-way ANOVAs followed by Tukey's multiple comparison test with a 95% confidence interval (*: p≤0.05, **: p≤0.01). Data shown are pooled from two separate experiments.

### Flow cytometry analysis of mouse vaginal cells

Local cell recruitment following vaginal administration of CpG ODN or α-GalCer was examined by flow cytometry. Mouse vaginas were aseptically removed at 12 h, 48 h and 72 h following local administration of CpG ODN, α-GalCer or control diluents. Vaginas (4 mice per group) were pooled in PBS, cut into small pieces and incubated at 37°C on a magnetic stirrer for 30 min in an EDTA/DTT solution containing 1 mM EDTA (Merck KGaA), 1 mM DTT (Sigma-Aldrich), 10 mM HEPES (Sigma-Aldrich), 1 µg/ml gentamicin sulfate (Sigma-Aldrich), and 5% FCS (Sigma-Aldrich) in HBSS (Gibco Invitrogen Life Technologies). Tissues were then filtered through a 250-µm filter and further disrupted for 2 h on a magnetic stirrer at 37°C in a Liberase/DNase solution containing 0.6 mg/ml liberase (Roche), 0.1 mg/ml DNase I (Sigma-Aldrich), and 11 mM HEPES (Sigma-Aldrich) in Iscoves modified medium (Sigma-Aldrich). The remaining tissues were mashed and filtered through a 70-µm cell strainer (BD Bioscience) to remove epithelial cells. After this step, the cell suspensions were washed and counted.

Vaginal single cell suspensions of 100,000 cells/well were used. Following blocking with CD16/32 for 20 min, vaginal cells were stained for 20 min at 4°C with the following monoclonal antibodies: goat α-mouse Gr1-PE (RB6-8C5), MHC-II-FITC (M5/114.15.2), CD11c-PE (N418) and Cd11b-APC (M1/70) (eBioscience). Samples were run on a BD FACSCalibur using Cellquest Pro software (BD Bioscience). Analysis was performed with FlowJo 7.5 (Tree Star, Inc.). Gates were set using live leukocytes identified by forward, side scatter (FSC and SSC respectively) and negative 7AAD (eBioscience) staining.

## Results

### Experimental design

We devised an experiment to analyze genome-wide transcriptional alterations in mouse vaginas in response to vaginal administration of the experimental CpG ODN or α-GalCer adjuvants. Our previous studies showed that these adjuvants are equally potent in the female mouse genital tract [7], [8]. Four animals from each group were treated i.vag. with a single dose of either adjuvant or reagent diluents, which were PBS for the CpG ODN group and PBS/Tween 0.5% for the α-GalCer group. Then, the vaginas were excised and processed for RNA extraction at 4 h, 24 h and 48 h post-treatment. Individual RNA samples were then subjected to a whole mouse genome microarray expression analysis. Normalized microarray expression data were evaluated based on responses to the treatments by pair-wise comparisons between treated and control mice. This evaluation was done at each time point to avoid time difference effects. Integrated analyses were employed to identify the signature processes and pathways with respect to the individual adjuvant and for comparisons between the two adjuvants.

### Primary analysis of transcriptome data

**Figure 1:**
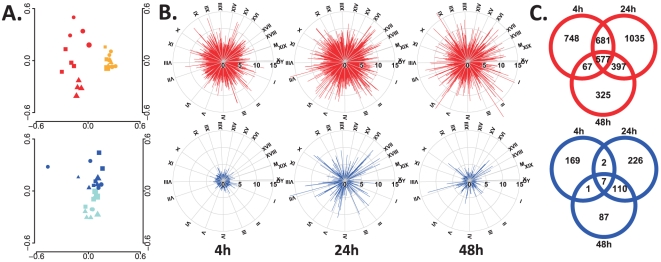
Transcriptome primary data analysis of mouse vagina following vaginal delivery of adjuvants. Gene expression in the vagina following CpG ODN (upper panel in red) or α-GalCer (lower panel in blue) delivery was monitored over time by microarray. Four biological replicates were independently analyzed for each time-point. A Following normalization, the quality of the data in terms of consistency was assessed by singular value decomposition. CpG ODN-treated mice (red) were compared to PBS controls (orange), and α-GalCer-treated mice (blue) were compared to PBS/Tween controls (turquoise). Time points are characterized by plot symbols: circle = 4 h, square = 24 h and triangle = 48 h. The figure shows two dimensions, and a third dimension is illustrated by the size of the plot symbol. B Circular mapping plots showing alterations in gene expression over time following adjuvant delivery on a chromosomal arrangement of transcripts based on Q-values on a log_10_ scale. C Venn diagram illustrating the number of genes that are significantly (Q≤0.001) differentially or commonly expressed by the adjuvants when compared to the respective controls at different time points.

We first performed an SVD of the transcriptome data to assess the quality of our microarray experiments. Consistent alterations in gene expression were noted among similarly treated mice when compared to their respective controls in two independent experiments. The largest separation of the data at each time point after treatment was observed for the CpG ODN group when compared to the α-GalCer group (Fig. 1A). A time-dependent pattern in the spread of the data was observed for the CpG ODN group, but only a minor difference in terms of time could be detected among animals given α-GalCer. Intra-group as well as inter-group data from the two differently treated control groups (PBS and PBS/Tween) showed little variation (Figure S1).

A cut-off Q-value of less than 3 on a log scale (Q≤0.001) was set for analysis. As depicted in Figure 1B-C, CpG ODN induced rapid alteration in the expression of 2,073 genes 4 h after administration. Alterations in gene expression peaked at 24 h, with 2,690 genes showing differential expression after CpG ODN administration, but at 48 h, only 1,366 genes were differentially expressed. A vast proportion of the significantly altered genes were altered at multiple time points, with the most shared genes observed at 4 h and 24 h. The kinetics of alterations in gene expression following α-GalCer administration showed similar patterns, although the overall response was weaker. Four hours after α-GalCer administration, 179 genes showed significantly altered expression, followed by 345 genes at 24 h and only 205 genes at 48 h. Several genes with significant change in expression following α-GalCer treatment were common between the different time points, with the most common genes (117) observed at 24 h and 48 h.

### Identification of top biological process engaged in response to adjuvants

**Figure 2:**
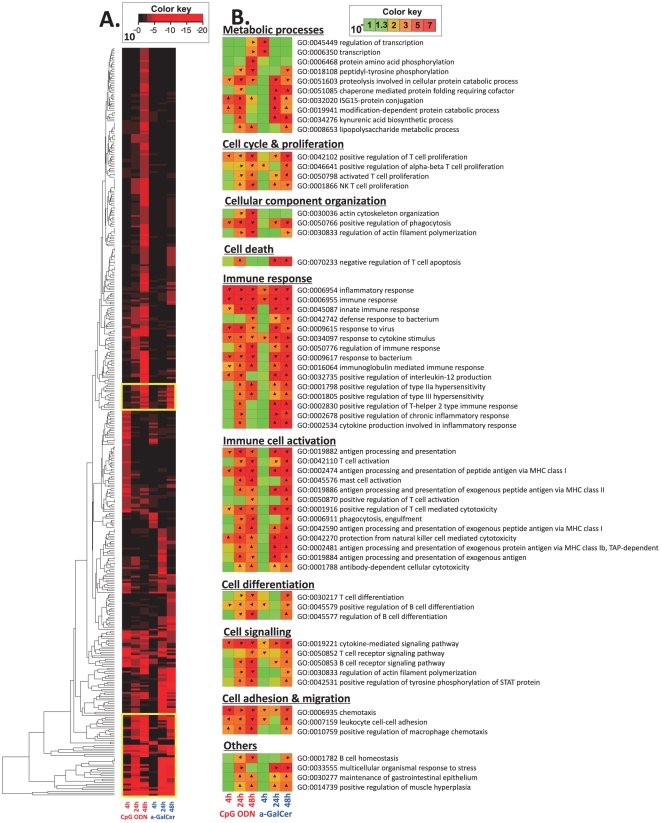
Biological processes analysis of significantly modulated genes identified in the mouse vagina in response to vaginal delivery of adjuvants. Up-regulated genes in the vagina at 4 h, 24 h and 48 h following local CpG ODN or α-GalCer administration were annotated with GO terms for biological processes. GO terms were selected for mapping if they were significant in either of the two adjuvant treated groups for at least one time point. A Color mapping and clustering of GO terms based on Q-values revealed distinct regions for exclusive and shared significantly expressed GO terms. B GO terms from the two regions showing high significance for both adjuvants were jointly grouped into wider bio-functional terms. Colors range from green to red, indicating increasing Q-value, and the angle of the arrows represents the proportion of genes within that GO term that are up- or down- regulated. A completely vertical arrow pointing upward indicates 100% of the genes annotated to that specific GO term were up-regulated, while a horizontal arrow means that 50% of the genes were up-regulated and that 50% of the genes were down-regulated.

To identify the main biological processes targeted by CpG ODN and α-GalCer, gene annotations were retrieved to identify GO terms. The level of significance of each GO term was evaluated using a reporter algorithm [19], [20] to map the GO terms (expressed as Q-values of transcriptome data). Hierarchical clustering based on significant Q-values of GO terms belonging to biological processes was performed to cluster response patterns induced by the two adjuvants (Fig. 2A and Figure S2).

Clustering GO terms for up-regulated genes allowed us to identify distinct regions with commonly and differentially expressed GO terms for the two adjuvants, as summarized in Fig. 2A and Figure S6. Two GO term regions showed high levels of significance for both CpG ODN and α-GalCer and were chosen for further analysis (Fig. 2A). GO terms from these common regions were grouped into broader terms for biological processes (Fig. 2B and Figure S6). Approximately half of the commonly up-regulated GO terms were grouped into either “immune response” or “immune cell activation” (Fig. 2B). The majority of genes within the highly significant GO term groups were up-regulated by both adjuvants for at least one time point. Common significantly up-regulated GO terms related to the immune system included “inflammatory response,” “cytokine production involved in inflammatory response,” “cytokine-mediated signaling pathway,” “response to cytokines stimulus,” and “chemotaxis” in addition to several GO terms related to ”immune response” and “antigen processing and presentation”. In addition, two regions containing GO terms significantly up-regulated at 4 h and 48 h were exclusively in the CpG ODN group (Figure S6A–B). At 4 h, the largest sorted GO term group up-regulated solely in the CpG ODN group was “cell signaling,” which contains “cell surface pattern recognition receptor signaling pathways” (Figure S6B). Importantly, several GO terms up-regulated at 48 h in the CpG ODN group included negative regulation of “toll-like receptor signaling pathway,” “innate immune response,” “chronic inflammatory response to antigenic stimulus” and “humoral immune response” (Figure S6A). Two selected regions were identified for α-GalCer (Figure S6C-D). At 4 h, the majority of GO terms exclusive to the α-GalCer group belonged to metabolic processes, while at 24 h, GO terms under “immune response” and “immune cell activation” were up-regulated (Figure S6C). Several GO terms exclusively up-regulated at 48 h after α-GalCer treatment involved T cell responses such as “T cell co-stimulation,” “positive regulation of CD4-positive, alpha beta T cell differentiation,” and “regulation of T cell receptor signaling pathway” (Figure S6D).

The majority of the significant GO terms in down-modulated genes were exclusive to the CpG ODN treated group and included “metabolic and cell cycle events,” “response to interferon gamma,” “negative regulation of inflammatory response to antigenic stimulus” and “positive regulation of myeloid cell differentiation.” α-GalCer induced down-regulation of several genes in the “metabolic processes and cell cycle events” GO term group (Figure S2A).

### Molecular pathway analysis

**Figure 3:**
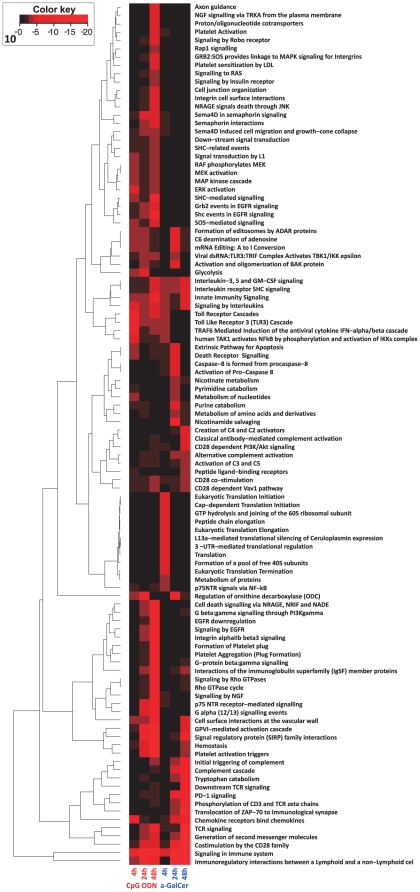
Adjuvant induced molecular pathways in the mouse vagina. Mapping and clustering of CpG ODN- or α-GalCer-induced pathways in the vagina based on Q-values. Pathways were selected for mapping if they demonstrated significance (Q≤0.001) in either of the two adjuvant-treated groups for at least one time point.

To understand the biological role of the differentially regulated genes, pathway analysis was performed. A number of pathways involved in innate immune response were identified among genes up-regulated by both adjuvants (Fig. 3). These pathways included “innate immune signaling,” “TLR cascades,” and “interleukin- and chemokine signaling.” Interestingly, several pathways involved in activation of classic and alternative complement pathways were modulated by α-GalCer. Only the “initial triggering of complement” term was identified in the CpG ODN group. Another intriguing finding was the activation of platelet associated pathways in the mouse vagina at 48 h after adjuvant treatment. Pathways for “platelet activation” and “platelet aggregation” were triggered in the mouse vagina 48 h after CpG ODN and α-GalCer administration. The majority of the pathways identified from down-modulated genes were involved in cell cycle events for both adjuvants and protein metabolism and lipid metabolism for CpG ODN and α-GalCer, respectively (Figure S3).

### Gene co-expression module analysis

**Figure 4:**
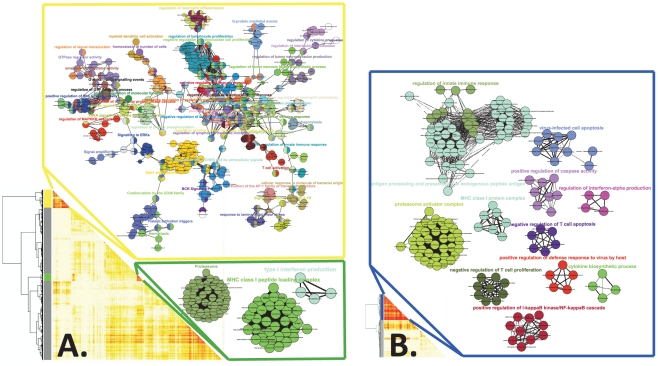
Co-expression and module network analysis of significantly altered genes significantly in the mouse vagina in response to local administration of adjuvants. Genes identified as having significantly altered expression, i.e., a Q-value of≤0.01 following A CpG ODN or B α-GalCer administration were analyzed for co-expression. Rows and columns of the heat map represent the same genes. The level of co-expression (connectivity) between genes is displayed by color intensity in the heat map, ranging from no connectivity in light yellow to a strong connection in red. High connectivity modules, shown as yellow, green and blue bars, were identified, and rectangular frames show functional terms and inter-relationships of annotated biological terms of the genes within each module. For a larger version of this figure, refer to Figure S7.

Several studies across a range of experimental conditions support the notion that coordinated behavior in gene expression indicates the presence of functional relationships between genes. Further, several studies confirmed the versatility of co-expression analysis for inferring gene functions, although co-expression does not necessarily represent a regulatory relationship [25]. To pinpoint correlation patterns among significantly altered transcripts in the mouse vagina in response to adjuvants, we examined co-expression of significantly altered gene transcripts (Q≤0.01) over time. Thus, a network construction program in which the highest inter-connected genes are clustered into forming modules was employed [21], and the results are summarized in Figure 4. Two significant gene co-expression modules containing approximately 3,600 genes were identified for the CpG ODN group (Fig. 4A). One significant gene co-expression module (Fig. 4B) containing approximately 700 genes was identified for the α-GalCer group. Functional terms were identified for genes within each module and graphically displayed as integrated networks. In the CpG ODN modules, the most connected functional groups include “regulation of cytokine production,” “regulation of neutrophil differentiation and chemotaxis,” “platelet activation triggers,” “regulation of lymphocyte proliferation,” “MHC class I peptide loading complex” and “proteasome.” In the α-GalCer module, the major connected functional groups were “regulation of innate immune response,” “antigen processing and presentation of endogenous peptide antigen” and “proteasome activator complex.” In addition, a number of smaller independent networks of functional groups were observed in the α-GalCer module (Fig. 4B).

### IPA revealed “inflammatory response” as the top biological function of both adjuvants

**Figure 5:**
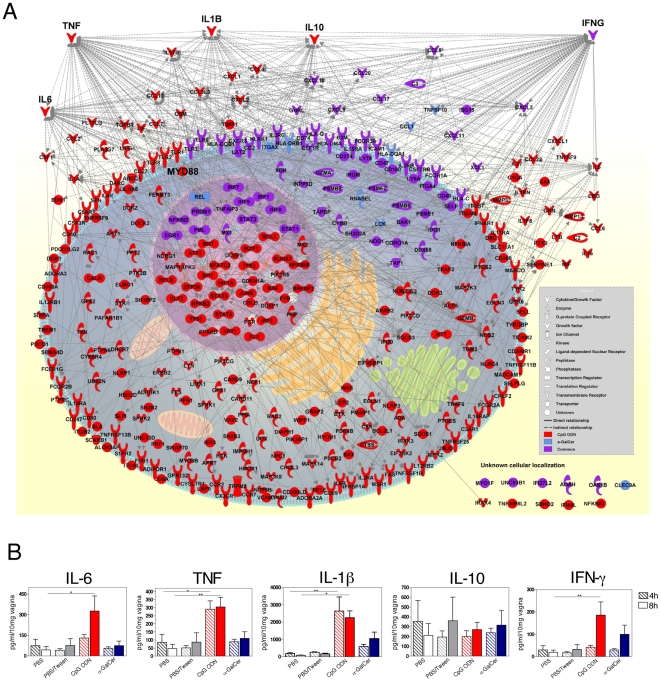
Adjuvant-induced inflammatory response bio-function in the mouse vagina. Following vaginal administration of CpG ODN or α-GalCer, Ingenuity Pathway Analysis identified “Inflammatory response” as the top bio-function network among significantly up-regulated (Q≤0.01) genes. A A network showing cellular localization and known relationships between genes involved in the inflammatory response. Genes are colored based on their induction by CpG (red), α-GalCer (blue) or both adjuvants (purple). B Key interactor genes are highlighted in A and were confirmed at the protein level by ELISA (IL-1β) and CBA (IL-6, TNF, IL-10 and IFN-γ). Results shown are pooled from two independent experiments. Bars show mean+SEM (*: p≤0.05, **: p≤0.01).

IPA was used to characterize the pattern of molecular interactions of significantly up-regulated transcripts (Q≤0.01) in the mouse vagina in response to adjuvants. “Inflammatory response” was identified as the top biological function at all studied time points following administration of either CpG ODN or α-GalCer. Networks illustrating the relationships between significantly up-regulated genes involved in the inflammatory response for each time point were generated and are illustrated in Figure S4. Complicated networks of approximately 200 significantly up-regulated genes involved in inflammatory response were seen in the CpG ODN group at each time point studied. α-GalCer generated a network of less than 50 genes, with relatively few relationships at each time point. To acquire an overall picture of the inter-related network of transcripts involved in inflammation, genes whose expression was significantly up-regulated in at least one of the three time points following adjuvant administration were considered for network analysis, and the results are summarized in Figure 5. A total of 345 gene transcripts were assigned to “inflammatory response.” Of these genes, 336 genes were targeted by CpG ODN, and 83 genes were targeted by α-GalCer. Approximately one-third of these molecules were localized to the cytoplasm, while another third were membrane/receptor bound (Fig. 5). The remaining genes were divided between the nucleus and extracellular regions, and the majority of genes were identified as transcriptional regulators or cytokine/chemokines. Within the inflammatory networks, significantly up-regulated transcripts with the most interactions with other significantly up-regulated transcripts, hereafter termed the main interactors, were identified for the 2 adjuvants. Interestingly, IFN-γ, a critical cytokine gene involved in concerted innate and adaptive immune responses, was identified as the main interactor transcript for both adjuvants. Protein expression of IFN-γ in the mouse vagina was confirmed 8 h after vaginal administration of either CpG ODN or α-GalCer (Fig. 5B). In addition, Tnf, Il-6, Il-1b, Il-10 and MyD88 were shown to serve as major interactors exclusively for CpG ODN. Protein expression levels of TNF-α, IL-1β and IL-6 were only significantly up-regulated in the CpG ODN-treated group when compared to the control group. However, no significant increase in IL-10 protein levels could be detected in any of the adjuvant-treated groups in comparison with their respective control groups (Fig. 5B). These results indicate that the two adjuvants can trigger up-regulation of genes involved in “inflammatory response.” This effect occurs, to a more limited extent, in response to α-GalCer, but Ifng serves as the main interactor of the inflammatory response network shared by both adjuvants.

## Discussion

This study strived to pinpoint signature molecules, pathways, and processes involved in innate immune responses in the mouse vagina following local administration of experimental mucosal adjuvants. CpG ODN and α-GalCer were selected for this study based on our previous findings that these adjuvants, when delivered i.vag. together with a HSV-2 gD protein, elicited comparable levels of protective immunity in the female genital tract against genital herpes [7], [8]. Complex vaginal tissues were used for analysis because they offer the unique advantage of monitoring alterations in gene expression related to multifaceted cell-to-cell interactions under physiological conditions.

The kinetics and magnitude of the overall alterations in gene expression differed between the two types of adjuvants. While CpG ODN induced a quick change in expression of 3,830 genes, with 1,366 genes remaining elevated at 48 h, the response to α-GalCer was more modest and transient, with a total of 602 genes modulated over all studied time points (Fig. 1B and C).

In addition to innate immune responses and inflammatory responses, a number of biological processes associated with antigen processing and presentation and T cell activation that are important for initiating an adaptive immune response were commonly induced by the two adjuvants (Fig. 2B). Inflammatory response was identified by IPA as the main bio-function shared by both CpG ODN and α-GalCer (Fig. 5A and Figure S4). These results extend our previous real time RT-PCR results, in which the expression of more inflammatory cytokine and chemokine genes were more robustly expressed in the mouse vagina after local delivery of CpG ODN when compared to α-GalCer [13]. IPA has successfully been used to identify relationships and connections between differentially expressed genes. For example, IPA demonstrated the mechanisms of action of CpG ODN in mouse spleens and elucidated the mode of action of the human yellow fever vaccine [11], [24]. Although both adjuvants induced an inflammatory response, the characteristics of the responses differed. CpG ODN modulated the expression of approximately four times as many inflammatory genes when compared to α-GalCer (Fig. 5A). Ifng was the common interactor in the inflammatory responses elicited by both adjuvants, but Tnf was exclusively induced by CpG ODN (Fig. 5A). A recent microarray and bioinformatic study on gene expression identified Tnf and Ifng as the main inducers in the murine spleen following intra-peritoneal injection of CpG ODN [11].

Our co-expression studies identified networks of “regulation of neutrophils differentiation” and “positive regulation of neutrophil chemotaxis,” which contained highly inter-connected genes among CpG ODN-induced networks (Fig. 4A). The neutrophil-attracting ELR-CXC chemokines Cxcl1 and Cxcl2 genes were also highly up-regulated in the vagina of CpG ODN-treated mice (Fig. 5A). In line with this observation, our flow cytometry analysis could detect more than a ten-fold increase in the percentage of neutrophils (CD11b^+^, Gr-1^+^, MHC II) in the vagina 12 h after CpG ODN administration. Notably, the levels of neutrophil infiltration in the vagina were reduced at 48 h and returned to the same basal levels as the controls at 72 h (Figure S5). We also showed that the percentage of macrophages (MHC II^+^, CD11b^+^, CD11c^-^) and DCs (MHC II^+^, CD11b^+^, CD11c^+^) increased in the mouse vagina after administration of CpG ODN or α-GalCer. These findings are consistent with a previous study that showed expansion of tissue neutrophils, macrophages and DCs following mucosal CpG ODN delivery [26]. Whereas triggering an inflammatory response is considered to be required for adjuvants to exert their function, excessive inflammation may lead to serious tissue damage. In fact, our previous results demonstrate that CpG ODN, but not α-GalCer, causes massive inflammatory cell infiltration into the mouse vagina and leads to damage to the mouse vaginal epithelium [13].

We employed an integrated analysis of the transcriptome data to pinpoint signature pathways and processes that could not have been otherwise realized by standard clustering methods. This integrated approach has been recently used to identify the molecular effects of diet on metabolism in mice [27]. An intriguing finding that emerged from our integrated analysis was the co-expression of genes involved in proteasome activation and MHC class I peptide loading in the vagina of mice given CpG ODN or α-GalCer. Given the critical importance of proteasome and MHC class I loading events in the development of cytotoxic T cell CTL response [28], it is likely that these 2 adjuvants can trigger a CTL response following vaginal administration. Interestingly, we could show that female C57Bl/6 mice immunized i.vag. with the model antigen ovalbumin in combination with CpG ODN or α-GalCer (same doses of adjuvants used in the current study) developed a potent ovalbumin SIINFEKL peptide-specific *in vivo* CTL response in lymph nodes that drained the genital tract (Lindqvist M et al., un-published results).

Both common and different molecular pathways were triggered in the vagina in response to adjuvants. Thus, classical and alternative pathways of complement activation were induced by α-GalCer. However, the C3 molecule was significantly up-regulated in the vagina in response to both CpG ODN and α-GalCer. The involvement of the complement system in modulating T- and B cell responses by bridging innate and adaptive immunity has been previously established [29]. Interestingly, the C3 molecule has been shown to function as a molecular adjuvant to enhance humoral immune responses to co-administered antigens in mice [30]. Pathways involved in “platelet activation and aggregation” and “platelet activation triggers” were targeted by CpG ODN and α-GalCer, respectively. Platelets play an important role in inflammation in addition to their role in thrombosis [31]. Thus, through interactions with endothelial cells, platelets induce NF-κB signaling and enhance neutrophil migration into tissue [32], [33]. Notably, up-regulation in expression of genes involved in inhibitory receptor programmed death 1 (PD-1) signaling, which has an essential role in controlling excessive inflammatory response [34], was only observed in the vaginas of α-GalCer treated mice (Fig. 3). This expression pattern may explain the more controlled inflammatory response elicited in the vagina in response to α-GalCer. Another pathway induced only by CpG ODN was glycolysis. TLR agonists can induce glycolysis and thereby regulate DC activation [35]. Furthermore, the end product of glycolysis, lactate, is an important component in maintaining the acidic pH of the vagina; therefore, it contributes to the innate defenses of the female genital tract [36].

In conclusion, through employment of integrated analyses of transcriptome data, we identified signature pathways, processes and networks shared by two classes of promising mucosal adjuvants, namely the TLR9 agonist CpG ODN and the iNKT cell agonist α-GalCer. To our knowledge, this is the first integrated genome-wide transcriptome analysis on the effect of immunomodulatory adjuvants on immunological functions and pathways in the mammalian female genital tract. These results provide new insights into understanding the mechanism of action of mucosal adjuvants in the female genital tract and therefore may inform rational development of effective vaccine adjuvant and immunotherapeutic approaches to counter STIs.

## Supporting Information

Figure S1 **Singular value decomposition (SVD) analysis for validation of microarray data.** Alterations in gene expression in the vagina following administration of CpG ODN (red), α-GalCer (blue) or the PBS (orange) and PBS/Tween (turquoise) controls were monitored over time by microarray. Following normalization, the quality of the data in terms of consistency was assessed by SVD. Time points are characterized by the following plot symbols: circle = 4 h, square = 24 h and triangle = 48 h. The figure shows two dimensions, and a third dimension is illustrated by the size of the plot symbol.
(EPS)

Figure S2 **Annotation and expression of biological processes.** Down-regulated genes in the vagina at 4 h, 24 h and 48 h following CpG ODN or α-GalCer administration were annotated to GO terms for biological processes. GO terms that were significant (Q≤0.001) for either adjuvant and any time point were color mapped and clustered based on Q-values.
(EPS)

Figure S3 **Down-modulated pathways in the murine vagina in response to vaginal delivery of adjuvants.** Mapping and clustering of CpG ODN or α-GalCer down-modulated pathways in the vagina based on Q-values. Pathways were selected for mapping if they demonstrated significance (Q≤0.001) in either of the two adjuvant-treated groups for at least one time point.
(EPS)

Figure S4 **Inflammatory networks and major interactor genes identified in the murine vagina in response to vaginal delivery of adjuvants.** Ingenuity Pathway Analysis identified “Inflammatory response” as a top bio-function following vaginal administration of CpG ODN or α-GalCer in the mouse vagina. Networks show relationships between significantly up-regulated genes (Q≤0.01) at each time-point, where full lines are direct relationships and dotted lines indicate indirect relationships. Color labels ranging from bright pink to red are representative of the degree of the increase in fold change expression values compared to the respective control groups (darker reds represent higher gene expression levels).
(EPS)

Figure S5 **Recruitment of innate immune cells to the mouse vagina following vaginal administration of adjuvants.** Groups of mice were i.vag. administered a single dose of CpG ODN, α-GalCer, PBS or PBS/Tween 5%. After 12 h, 48 h and 72 h following vaginal delivery of CpG ODN or α-GalCer, vaginas were excised and pooled, and cells were extracted. Cells were stained for cell markers, and flow cytometry was used to quantify the percentage of: **A and C** neutrophils expressing CD11b^+^, Gr-1^+^, and MHC II^-^; **A and E** DC-like cells expressing CD11b^+^, Gr-1^+^, and MHC II^+^; **B and D** macrophages, identified as MHC II^+^, CD11b^+^, CD11c^-^ cells; and **B and F** conventional DCs, identified as being MHC II^+^, CD11b^+^, and CD11c^+^. Gates were set on live leukocytes, and the plots in the lower rows of A and B were gated on the double positive cells in the upper row. Plots shown are representative of 12 h post-treatment from two independent experiments with pooled samples from 4 mice in each group and time point. Numbers represent the percentage of the gated population, while numbers in parenthesis in the lower row show abundance of live leukocytes.
(EPS)

Figure S6 **Grouping of biological processes identified in the murine vagina in response to local administration of adjuvants.** Broader functional grouping of GO terms for significantly induced biological processes by CpG ODN and α-GalCer. Panels **A** and **B** display CpG ODN-specific GO terms with the highest significance at 48 h and 4 h, while **C** and **D** are α-GalCer specific. Colors range from green to red to indicate increasing Q-value, and the angle of the arrows represents the proportion of genes within that GO term that are up- or down-regulated. A completely vertical arrow pointing upward indicates 100% of the genes annotated with that specific GO term were up-regulated, while a horizontal arrow means that 50% of the genes were up-regulated.
(EPS)

Figure S7 **Co-expression and module network analysis of significantly altered genes significantly in the mouse vagina in response to local administration of adjuvants.** Genes identified as having significantly altered expression, i.e., a Q-value of#0.01 following A CpG ODN orB a-GalCer administration were analyzed for co-expression. Rows and columns of the heat map represent the same genes. The level of cohyphen;expression (connectivity) between genes is displayed by color intensity in the heat map, ranging from no connectivity in light yellow to a strong connection in red. High connectivity modules, shown as yellow, green and blue bars, were identified, and rectangular frames show functional terms and interrelationships of annotated biological terms of the genes within each module. This supporting figure is a larger version of Figure 4.
(PDF)

Table S1 **List of Q-values, log 2 fold changes, the pathway and GO networks including their associated gene members used for the integration analysis of the transcriptomic data.**
(XLSX)

## References

[1] Stanberry LR (2004). Clinical trials of prophylactic and therapeutic herpes simplex virus vaccines.. Herpes 11 Suppl.

[2] Tramont EC (1989). Gonococcal vaccines.. Clin Microbiol Rev 2 Suppl.

[3] Mestecky J, Fultz PN (1999). Mucosal immune system of the human genital tract.. J Infect Dis.

[4] Russell MW (2002). Immunization for protection of the reproductive tract: a review.. Am J Reprod Immunol.

[5] Kwissa M, Kasturi SP, Pulendran B (2007). The science of adjuvants.. Expert Rev Vaccines.

[6] Harandi AM, Davies G, Olesen OF (2009). Vaccine adjuvants: scientific challenges and strategic initiatives.. Expert Rev Vaccines.

[7] Tengvall S, Lundqvist A, Eisenberg RJ, Cohen GH, Harandi AM (2006). Mucosal administration of CpG oligodeoxynucleotide elicits strong CC and CXC chemokine responses in the vagina and serves as a potent Th1-tilting adjuvant for recombinant gD2 protein vaccination against genital herpes.. J Virol.

[8] Lindqvist M, Persson J, Thorn K, Harandi AM (2009). The mucosal adjuvant effect of alpha-galactosylceramide for induction of protective immunity to sexually transmitted viral infection.. J Immunol.

[9] Klaschik S, Gursel I, Klinman DM (2007). CpG-mediated changes in gene expression in murine spleen cells identified by microarray analysis.. Mol Immunol.

[10] Kato A, Homma T, Batchelor J, Hashimoto N, Imai S (2003). Interferon-alpha/beta receptor-mediated selective induction of a gene cluster by CpG oligodeoxynucleotide 2006.. BMC Immunol.

[11] Klaschik S, Tross D, Klinman DM (2009). Inductive and suppressive networks regulate TLR9-dependent gene expression in vivo.. J Leukoc Biol.

[12] Mosca F, Tritto E, Muzzi A, Monaci E, Bagnoli F (2008). Molecular and cellular signatures of human vaccine adjuvants.. Proc Natl Acad Sci U S A.

[13] Lindqvist M, Navabi N, Jansson M, Samuelson E, Sjoling A (2009). Local cytokine and inflammatory responses to candidate vaginal adjuvants in mice.. Vaccine.

[14] Hubbell E (2005). Plier White Paper.. Santa Clala.

[15] Bolstad BM, Irizarry RA, Astrand M, Speed TP (2003). A comparison of normalization methods for high density oligonucleotide array data based on variance and bias.. Bioinformatics.

[16] Benjamini Y, Drai D, Elmer G, Kafkafi N, Golani I (2001). Controlling the false discovery rate in behavior genetics research.. Behav Brain Res.

[17] Ashburner M, Ball CA, Blake JA, Botstein D, Butler H (2000). Gene ontology: tool for the unification of biology. The Gene Ontology Consortium.. Nat Genet.

[18] Croft D, O'Kelly G, Wu G, Haw R, Gillespie M Reactome: a database of reactions, pathways and biological processes.. Nucleic Acids Res.

[19] Patil KR, Nielsen J (2005). Uncovering transcriptional regulation of metabolism by using metabolic network topology.. Proc Natl Acad Sci U S A.

[20] Oliveira AP, Patil KR, Nielsen J (2008). Architecture of transcriptional regulatory circuits is knitted over the topology of bio-molecular interaction networks.. BMC Syst Biol.

[21] Zhang B, Horvath S (2005). A general framework for weighted gene co-expression network analysis.. Stat Appl Genet Mol Biol.

[22] Bindea G, Mlecnik B, Hackl H, Charoentong P, Tosolini M (2009). ClueGO: a Cytoscape plug-in to decipher functionally grouped gene ontology and pathway annotation networks.. Bioinformatics.

[23] Shannon P, Markiel A, Ozier O, Baliga NS, Wang JT (2003). Cytoscape: a software environment for integrated models of biomolecular interaction networks.. Genome Res.

[24] Querec TD, Akondy RS, Lee EK, Cao W, Nakaya HI (2009). Systems biology approach predicts immunogenicity of the yellow fever vaccine in humans.. Nat Immunol.

[25] Stuart JM, Segal E, Koller D, Kim SK (2003). A gene-coexpression network for global discovery of conserved genetic modules.. Science.

[26] Sajic D, Patrick AJ, Rosenthal KL (2005). Mucosal delivery of CpG oligodeoxynucleotides expands functional dendritic cells and macrophages in the vagina.. Immunology.

[27] Nookaew I, Gabrielsson BG, Holmang A, Sandberg AS, Nielsen J Identifying molecular effects of diet through systems biology: influence of herring diet on sterol metabolism and protein turnover in mice.. PLoS One.

[28] Benham AM, Neefjes JJ (1997). Proteasome activity limits the assembly of MHC class I molecules after IFN-gamma stimulation.. J Immunol.

[29] Morgan BP, Marchbank KJ, Longhi MP, Harris CL, Gallimore AM (2005). Complement: central to innate immunity and bridging to adaptive responses.. Immunol Lett.

[30] Dempsey PW, Allison ME, Akkaraju S, Goodnow CC, Fearon DT (1996). C3d of complement as a molecular adjuvant: bridging innate and acquired immunity.. Science.

[31] Wagner DD, Burger PC (2003). Platelets in inflammation and thrombosis.. Arterioscler Thromb Vasc Biol.

[32] Lam FW, Burns AR, Smith CW, Rumbaut RE (2010). Platelets enhance neutrophil transendothelial migration via P-selectin glycoprotein ligand-1.. Am J Physiol Heart Circ Physiol.

[33] Gawaz M, Page S, Massberg S, Nothdurfter C, Weber M (2002). Transient platelet interaction induces MCP-1 production by endothelial cells via I kappa B kinase complex activation.. Thromb Haemost.

[34] Brown KE, Freeman GJ, Wherry EJ, Sharpe AH Role of PD-1 in regulating acute infections.. Curr Opin Immunol.

[35] Krawczyk CM, Holowka T, Sun J, Blagih J, Amiel E Toll-like receptor-induced changes in glycolytic metabolism regulate dendritic cell activation.. Blood.

[36] Linhares IM, Summers PR, Larsen B, Giraldo PC, Witkin SS Contemporary perspectives on vaginal pH and lactobacilli.. Am J Obstet Gynecol.

