# Supplementary material for: Unraveling Molecular Signatures of Immunostimulatory Adjuvants in the Female Genital Tract through Systems Biology
Source: PLoS One. 2011 Jun 7;6(6):e20448. doi: 10.1371/journal.pone.0020448 (PMC3110186; doi:10.1371/journal.pone.0020448)
Supplement: Figure S7 — Co-expression and module network analysis of significantly altered genes significantly in the mouse vagina in response to local administration of adjuvants. Genes identified as having significantly altered expression, i.e., a Q-value of#0.01 following A CpG ODN orB a-GalCer administration were analyzed for co-expression. Rows and columns of the heat map represent the same genes. The level of cohyphen;expression (connectivity) between genes is displayed by color intensity in the heat map, ranging from no connectivity in light yellow to a strong connection in red. High connectivity modules, shown as yellow, green and blue bars, were identified, and rectangular frames show functional terms and interrelationships of annotated biological terms of the genes within each module. This supporting figure is a larger version of Figure 4. (PDF) [file pone.0020448.s007.pdf]

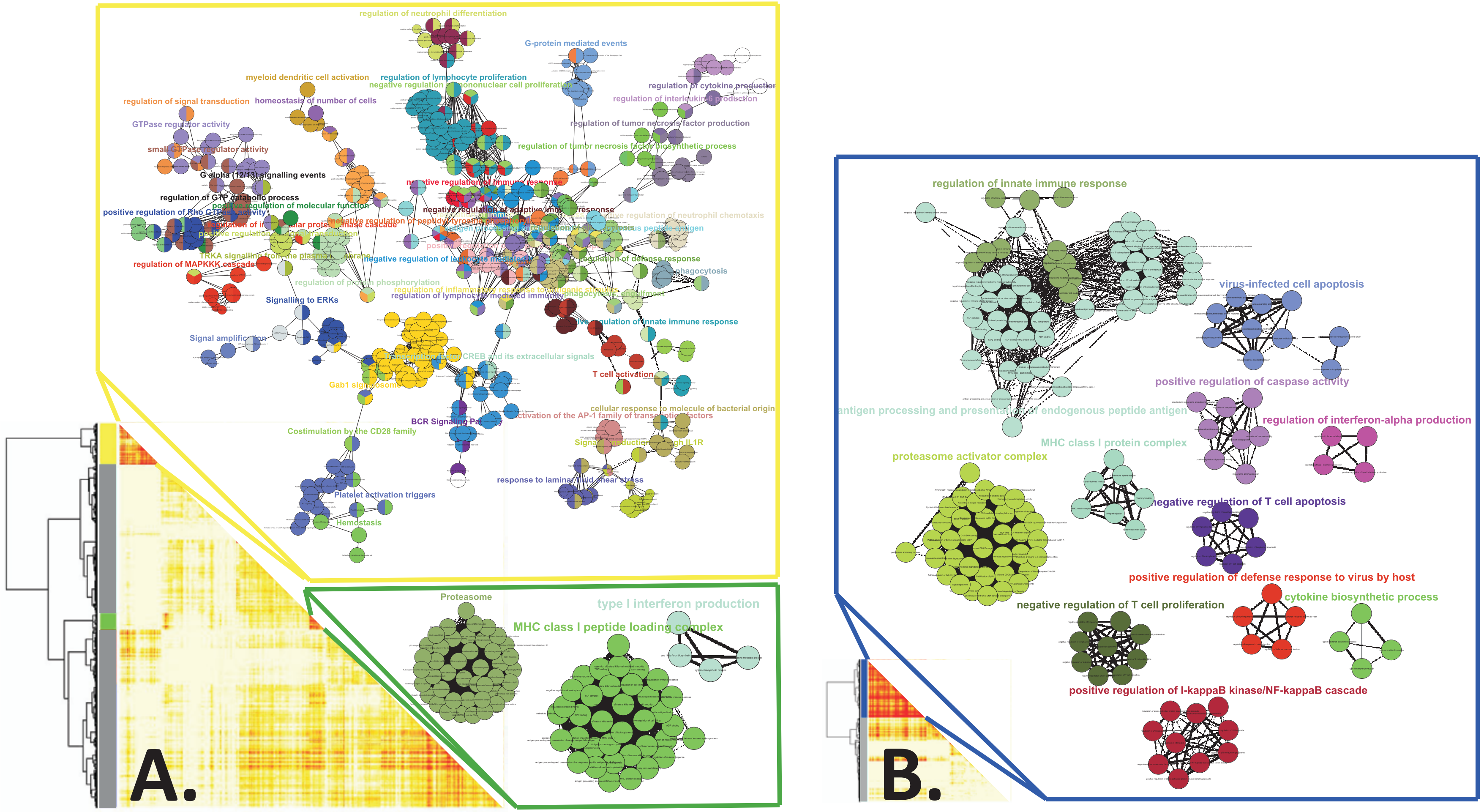

regulation of neutrophil differentiation
G-protein mediated events
myeloid dendritic cell activation
regulation of lymphocyte proliferation
regulation of signal transduction
homeostasis of number of cells
regulation of cytokine production
GTPase regulator activity
regulation of tumor necrosis factor production
small GTPase regulator activity
G alpha (12/13) signalling events
regulation of GTP catabolic process
positive regulation of Rho GTPase activity
regulation of MAPKKK cascade
regulation of protein phosphorylation
Signalling to ERKs
T cell activation
cellular response to molecule of bacterial origin
Costimulation by the CD28 family
Platelet activation triggers
Hemostasis
response to laminar fluid shear stress
Proteasome
type I interferon production
MHC class I peptide loading complex
A.
regulation of innate immune response
virus-infected cell apoptosis
positive regulation of caspase activity
regulation of interferon-alpha production
MHC class I protein complex
proteasome activator complex
negative regulation of T cell apoptosis
positive regulation of defense response to virus by host
negative regulation of T cell proliferation
cytokine biosynthetic process
positive regulation of I-kappaB kinase/NF-kappaB cascade
B.
